# Pheophytin a Inhibits Inflammation via Suppression of LPS-Induced Nitric Oxide Synthase-2, Prostaglandin E2, and Interleukin-1β of Macrophages

**DOI:** 10.3390/ijms151222819

**Published:** 2014-12-09

**Authors:** Chun-Yu Lin, Chien-Hsing Lee, Yu-Wei Chang, Hui-Min Wang, Chung-Yi Chen, Yen-Hsu Chen

**Affiliations:** 1 Division of Infectious Diseases, Department of Internal Medicine, Kaohsiung Medical University Hospital, Kaohsiung 807, Taiwan; E-Mail: infectionman@gmail.com; 2 School of Medicine, Graduate Institute of Medicine, Sepsis Research Center, College of Medicine, Kaohsiung Medical University, Kaohsiung 807, Taiwan; E-Mail: golden3p@gmail.com; 3 Research Center for Environmental Medicine, Kaohsiung Medical University, Kaohsiung 807, Taiwan; 4 Department of Nursing, Min-Hwei Junior College of Health Care Management, Tainan 736, Taiwan; E-Mail: chlee0818@gmail.com; 5 Department of Fragrance and Cosmetic Science, Kaohsiung Medical University, Kaohsiung 807, Taiwan; E-Mail: davidw@kmu.edu.tw; 6 Graduate Institute of Natural Products, Kaohsiung Medical University, Kaohsiung 807, Taiwan; 7 School of Medical and Health Sciences, Fooyin University, Kaohsiung 831, Taiwan; 8 Department of Biological Science and Technology, College of Biological Science and Technology, National Chiao Tung University, Hsinchu 300, Taiwan

**Keywords:** pheophytin a, NO, signal transducers and activators of transcription 1 (STAT-1), anti-inflammation

## Abstract

Inflammation is a serious health issue worldwide that induces many diseases such as sepsis. There has been a vast search for potentially effective drugs to decrease mortality from sepsis. Pheophytin a is a chlorophyll-related compound derived from green tea. We found that pre-treatment with pheophytin a suppressed lipopolysaccharide (LPS)-induced nitric oxide (NO), prostaglandin E2 (PGE2), and interleukin-1β in RAW 264.7 macrophages. NO synthase-2 (NOS2) and cyclooxygenase-2 (COX-2) expression levels were repressed by pre-treatment with pheophytin a at both the transcriptional and translational levels. Pheophytin a inhibited NOS2 promoter activity, but not its mRNA stability, through extracellular signal-regulated kinase (ERK1/2). This suppression was reversed by ERK1/2 inhibitor (U0126). Pheophytin a reduced signal transducers and activators of transcription 1 (STAT-1) activation, without an obvious influence on activator protein-1 (AP-1) and nuclear factor κB (NF-κB). These results suggest that pheophytin a functions by down-regulating the transcriptional levels of inflammatory mediators and blocking the ERK and STAT-1 pathways.

## 1. Introduction

Sepsis is a critical syndrome associated with microorganism infection that has a high mortality rate [1]. Although the “germ theory” was proposed several decades ago, host immune factors have recently been considered to have a more important role in the progression of sepsis [2]. Host immune response plays an important role when encountering microorganisms, and septic patients are considered to elevate immune response, leading to organ failure and death [3]. Agents developed to address the immune response during sepsis, such as CytoFab (a polyclonal anti-tumor necrosis factor antibody), have failed to improve the outcomes [2]. Hence, there is still much uncertainty on immune modulation during sepsis. In addition to the discovery of new antimicrobial agents, the development of new agents focusing on “immunomodulation” during sepsis is also urgent.

Gram-negative bacteria-induced bacteremia is common among patients with sepsis [4]. Lipopolysaccharides (LPS), a part of the cell walls of gram-negative bacteria and known as endotoxins [5], play an important role in sepsis and endotoxemia [6]. LPS will stimulate nitric oxide (NO), prostaglandin E2 (PGE2) overproduction and the release of inflammatory cytokines by macrophages, including interleukins (IL-1β, or IL-6) and tumor necrosis factor α (TNF-α) [7,8]. With LPS stimulation, macrophage recruitment/migration and the production of the above pro-inflammatory factors play key roles in sepsis severity and outcome.

Nuclear factor κB (NF-κB) signaling is an important mediator of the inflammatory response, cellular proliferation, and cell adhesion. NF-κB activation is controlled by the IκB kinase (IKK) complex, resulting in IκB degradation through the ubiquitin-proteasome system [9]. Subsequently, free NF-κB translocates to the nucleus and binds to specific binding sites in the promoter regions of its target genes, such as inducible nitric oxide synthase (iNOS, also named NOS2) and cyclooxygenase-2 (COX-2) [10]. The transcriptional activation of NF-κB is related to the phosphorylation of the mitogen-activated protein kinases (MAPKs) and pro-inflammatory cytokines in LPS-induced macrophages [11]. MAPKs, including c-Jun NH_2_-terminal kinase (JNK), extracellular signal-regulated kinase (ERK) and p38, are involved in the expression of pro-inflammatory genes during the LPS-induced inflammation process [12]. The phosphoinositide-3-kinase (PI3K)/Akt signaling pathway plays an important role in negatively regulating LPS-induced acute inflammatory responses *in vitro* and *in vivo* [13]. Inhibition of the PI3K/Akt signaling pathway can enhance the activation of NF-κB transcription factors and iNOS and COX-2 expression in RAW 264.7 cells [14]. In addition, the LPS-induced activation of transcription factors such as NF-κB and signal transducer and activator of transcription-1 (STAT-1) play pivotal roles in the expression of genes such as iNOS, COX-2 and inflammatory cytokines [15].

Pheophytin a (C_55_H_74_N_4_O_5_), a chlorophyll-related compound [16], is an important component of green tea. It has anti-inflammatory [17], anti-oxidant, and anti-chemotactic properties in human polymorphonuclear neutrophils [18], and anti-hepatitis C activity and functions in anti-tumor promotion. Because sepsis is a dysregulated inflammatory response, drugs that could modulate the production of inflammatory mediators might be useful to treat inflammatory diseases and endotoxin shock. These results have led us to hypothesize that pheophytin a might modulate NO, PGE2, and IL-1β production and the migration of macrophages during LPS-induced sepsis, and these issues are addressed in the present study. Hopefully the findings here might lead to the further development of new potential therapeutic or prophylactic agents for sepsis.

## 2. Results and Discussion

### 2.1. Cytotoxicity of Pheophytin a on Lipopolysaccharides (LPS)-Stimulated Macrophages

**Figure 1:**
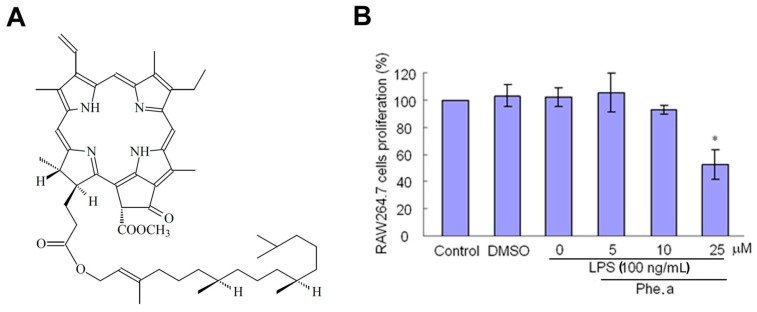
Effect of pheophytin a (Phe. a) treatment on RAW264.7 cells stimulated with lipopolysaccharides (LPS). (**A**) Chemical structure of pheophytin a; (**B**) Cells were pre-treated with pheophytin a for 30 min at various concentrations (5, 10 and 25 μM) before being stimulated with LPS (100 ng/mL) for 24 h and measured using an Alamar Blue assay. Data are expressed as the means ± standard deviations (SD) of three independent experiments. * *p* < 0.05 *vs.* control, by Student’s *t* test, *n* = 3–5.

Firstly, we evaluated the cell viability of RAW264.7 cells with LPS stimulation and pheophytin a (Figure 1A) at different dosages. Figure 1B shows that pheophytin a has cytotoxicity for LPS-stimulated RAW264.7 cells at 25 μM. In the present study, we performed the experiments using 10 μM pheophytin a.

**Figure 2:**
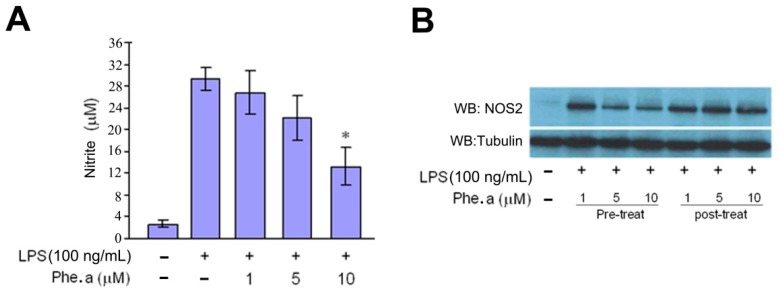

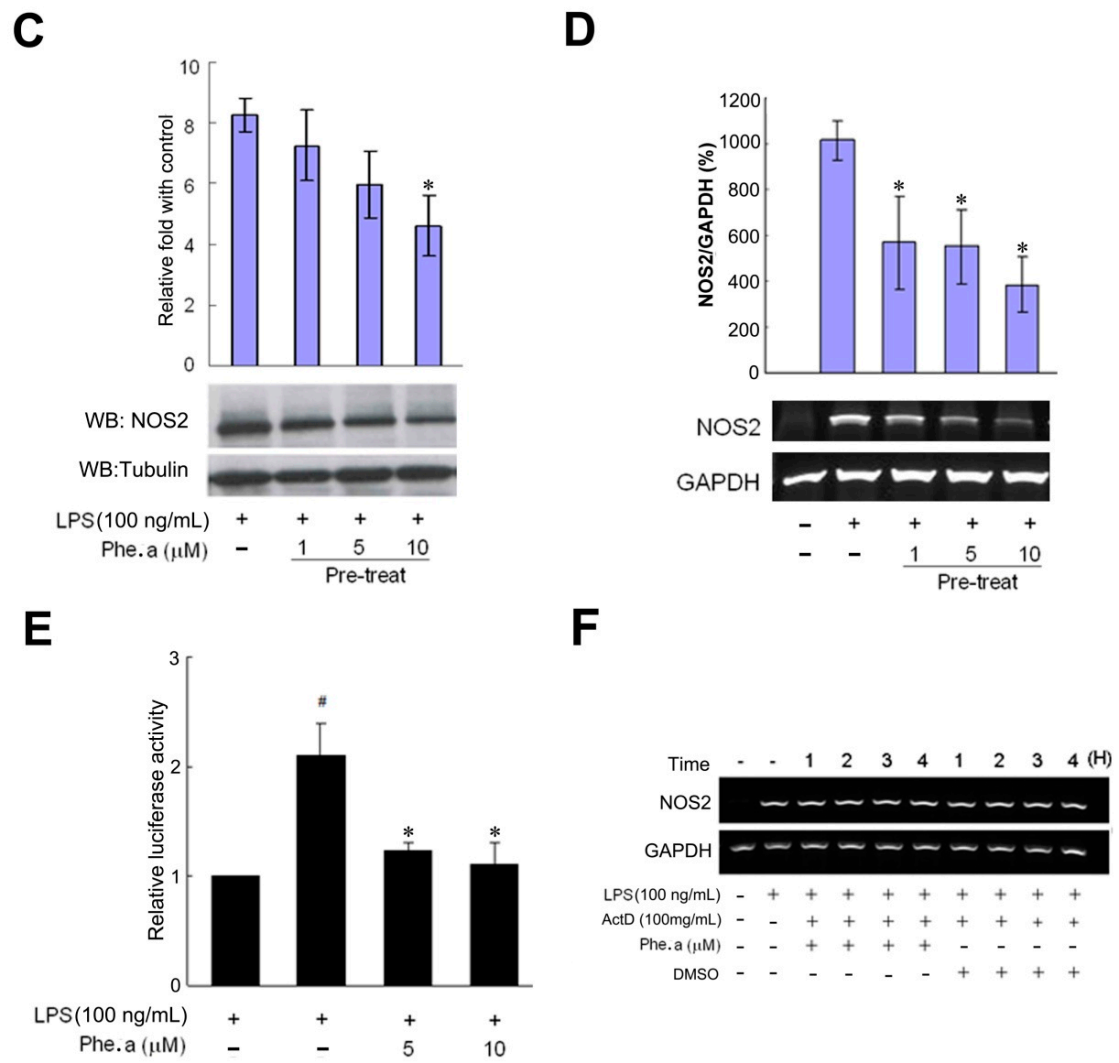
Effects of pheophytin a (Phe. a) on LPS-induced NOS2 production in RAW 264.7 cells. (**A**) Quantification of NO levels in pheophytin a-treated LPS-stimulated RAW 264.7 cells. Cells were treated with indicated concentrations of pheophytin a for 30 min, and NO production was quantified as described in the Section 3.7; (**B**) RAW 264.7 cells were pre-treated with the indicated concentrations of pheophytin a for 30 min followed by LPS (100 ng/mL) for 6 h. The pre-treatment with pheophytin a exerted significant repression of LPS-induced NOS2 protein production. In contrast, RAW 264.7 cells were post-treated with the indicated concentrations of pheophytin a (30 min after the LPS 100 ng/mL administration) and showed no suppression effect. Western blot analysis of NOS2 expression. *Tubulin* was used as a control gene; (**C**) RAW 264.7 cells were pre-treated with the indicated concentrations of pheophytin a for 30 min followed by LPS (100 ng/mL) for 6 h. Western blot analysis of NOS2 expression. *Tubulin* was used as a control gene. Quantification data are shown in the upper panel. The results are expressed as the ratio to control group (without LPS stimulation); (**D**) RAW 264.7 cells were treated with the indicated concentrations of pheophytin a for 30 min followed by LPS (100 ng/mL) for 3 h. RT-PCR analysis of NOS2 expression. *GAPDH* was used as a control gene. Quantification data are shown in the upper panel; (**E**) RAW264.7 cells were transiently transfected with NOS2 luciferase reporter constructs. 24 h after transfection, the cells were treated with pheophytin a for 30 min prior to stimulation with LPS (100 ng/mL) for 6 h before measurement of the luciferase activity. The relative luciferase activities are shown as percentages of the activity in cells stimulated with LPS; and (**F**) RAW264.7 cells were treated with Actinomycin D (an RNA synthesis inhibitor) or pheophytin a for 30 min prior to LPS stimulation (100 ng/mL) for 6 h before the analysis of the NOS2 mRNA levels by qPCR. Results show the means ± SD of three experiments. * *p* < 0.05 *vs.* LPS treatment and ^#^ *p* < 0.01 *vs.* control treatment, by Student’s *t* test, *n* = 3–6. These experiments were repeated three times with similar results.

### 2.2. Effects of Pheophytin a on Nitric Oxide (NO) Production in LPS-Stimulated Macrophages

The excessive NO production after infection, which is caused by iNOS, encoded by the *NOS2* gene [19], has been proposed to be the major factor of the tissue injury in septic shock [20]. Pre-treatment with pheophytin a for 30 min exerted significant repression of LPS-induced NO production in a dose-dependent manner (Figure 2A). In contrast, no suppression effect was noted in the group with pheophytin a post-treatment after LPS administration (Figure 2B). The LPS-induced expression of the *NOS2* gene was also repressed by pre-treatment with pheophytin a in a dose-dependent manner, including its protein (Figure 2C) and mRNA levels (Figure 2D). Our results suggest that pheophytin a significantly suppresses NOS2 expression at both the transcriptional and translational levels in LPS-stimulated RAW264.7 cells. To clarify the repression mechanisms of pheophytin a on NOS2, we transfected a luciferase reporter construct with the NOS2 promoter to determine NOS2 promoter activity under pheophytin a and/or LPS treatment. Compared to the LPS-treated group, pre-treatment with pheophytin a at both 5 and 10 μM effectively reduced the activity of the NOS2 promoter in LPS-stimulated RAW264.7 cells (Figure 2E). However, NOS2 mRNA stability was not affected by the pre-application of pheophytin a (Figure 2F).

### 2.3. Effects of Pheophytin a on Prostaglandin E2 (PGE2), Cyclooxygenase-2 (COX-2) and Interleukin-1β (IL-1β) Production in LPS-Stimulated Macrophages

**Figure 3:**
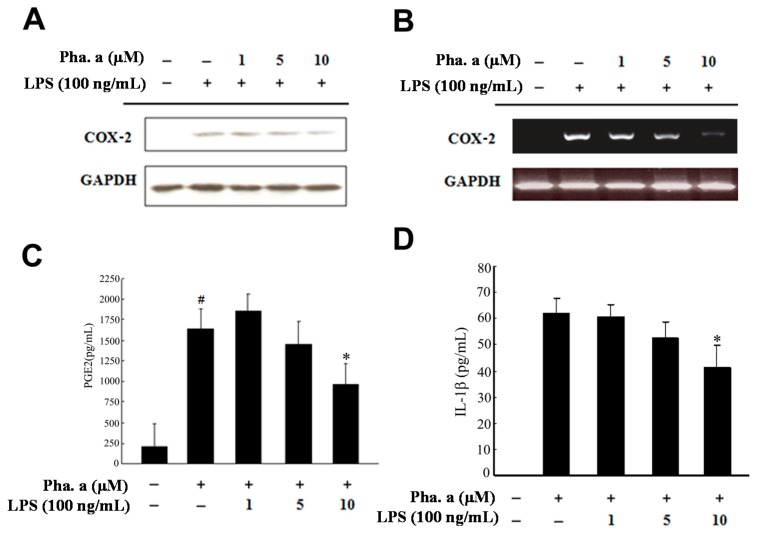
Effects of pheophytin a (Phe. a) on COX-2, PGE2 and IL-1β expression and production in LPS-stimulated RAW 264.7 cells. (**A**) RAW 264.7 cells were treated with the indicated concentrations of pheophytin a for 30 min followed by LPS (100 ng/mL) for 6 h. Western blot analysis of COX-2 expression. The intensity of the COX-2 band was normalized to *GAPDH* and is relative to the control; (**B**) RAW 264.7 cells were treated with the indicated concentrations of pheophytin a for 30 min followed by LPS (100 ng/mL) for 3 h. RT-PCR analysis of COX-2 expression. Intensity of the COX-2 band was normalized to GAPDH and is relative to the control; (**C**) RAW 264.7 cells were treated with the indicated concentrations of pheophytin a for 30 min followed by LPS (100 ng/mL). PGE2 assay analysis of PGE2 production; (**D**) Cells were pre-treated with various concentrations (1, 5 and 10 μM) of pheophytin a for 30 min before stimulation with LPS (100 ng/mL) for 24 h. The IL-1β concentrations were measured using ELISA kits. Results show the means ± SD of three experiments. * *p* < 0.01 *vs.* LPS treatment and ^#^ *p* < 0.001 *vs.* control treatment, by Student’s *t* test, *n* = 3–6. These experiments were repeated three times with similar results.

We further observed that both the protein and mRNA levels of COX-2 were also suppressed by Pheophytin a in a dose-dependent manner (Figure 3A,B). PGE2 is mainly synthesized by the COX-2 enzyme, which is also responsible for inflammatory symptoms [21]. Using an enzyme-linked immunosorbent assay (ELISA) kit, pheophytin a clearly attenuated the LPS-stimulated PGE2 production of RAW264.7 cells (Figure 3C). Moreover, IL-1β, a well-known proinflammatory cytokine, contributes to the systemic inflammation during sepsis [22]. Our study also revealed that pheophytin a suppressed IL-1β in LPS-stimulated RAW264.7 cells in a dose-dependent manner (Figure 3D), demonstrating that pheophytin a at cytotoxic levels in our experiments suppressed LPS-induced inflammatory responses in macrophages by attenuating NO synthesis and cytokine production.

### 2.4. Effects of Pheophytin a on Signal Transduction Pathways in LPS-Stimulated Macrophages

**Figure 4:**
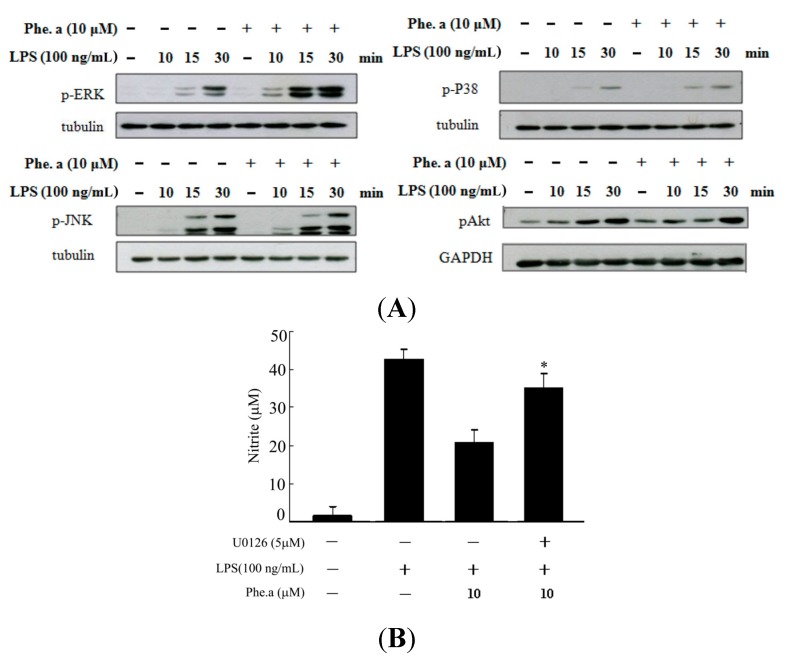
Effects of pheophytin a (Phe. a) on MAPK and Akt pathways in LPS-stimulated RAW 264.7 cells. (**A**) Western blot analysis of the phosphorylation of ERK, JNK, p38 and Akt expression induced by LPS. Cells were pre-treated with 10 μM pheophytin a for 30 min, and the total protein was harvested at three different time points (10, 15 and 30 min) after LPS stimulation (100 ng/mL); and (**B**) Pheophytin a suppressed LPS-induced NO production through ERK1/2 phosphorylation. Pre-treatment with U0126, a specific inhibitor of ERK1/2, partially blocked the inhibitory effect of pheophytin a on NOS2 expression and NO production, indicating a critical role for the MEK/ERK1/2 signaling pathway in the mechanism of pheophytin a. * *p* < 0.01 *vs.* pre-treat with pheophytin a on LPS-activated macrophages, by Student’s *t* test, *n* = 3–6. These experiments were repeated three times with similar results.

Several signal transduction pathways that regulate the production of pro-inflammatory cytokines and chemokines, such as MAPKs and Akt [22,23,24], are involved in sepsis. To further clarify the anti-inflammatory molecular mechanisms of pheophytin a, we investigated these signal transduction pathways, including p38, JNK and ERK. Our results showed that 10 μM pheophytin a significantly increased ERK1/2 in RAW264.7 cells with LPS administered, however, it had no effect on the expression of JNK, p38 and Akt (Figure 4A). To confirm whether the ERK1/2 pathway participates in the anti-inflammatory effect of pheophytin a in sepsis, a specific ERK1/2 inhibitor, U0126, was used. We found that pre-conditioning using U0126 partially reversed the NO production reduction effect caused by pheophytin a application (Figure 4B). The MAPK family plays an important role in the LPS-induced expression of iNOS, COX-2, and proinflammatory cytokines in many types of cells [25,26]. Several natural products inhibit the expression of these genes by modulating MAPK phosphorylation. These results indicate that the inhibitory effect of pheophytin a on LPS-activated macrophages occurs through ERK signaling pathways.

### 2.5. Effects of Pheophytin a on the Activation of NF-κB, AP-1 and STAT-1 in LPS-Stimulated Macrophages

**Figure 5:**
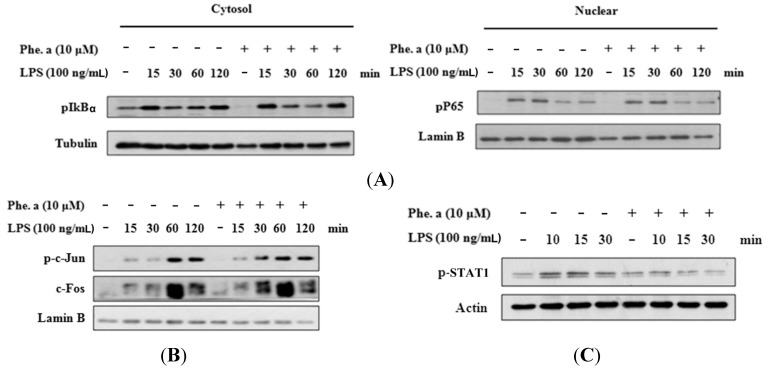
Effects of pheophytin a (Phe. a) on NF-κB, AP-1 and STAT-1 activation in LPS-stimulated RAW 264.7 cells. (**A**) Cytoplasmic (**Left**) or nuclear (**Right**) extracts prepared from cells treated with LPS were analyzed for levels of the signaling molecules leading to NF-κB α activation by Western blot analysis; (**B**) Detection of AP-1 subunits (c-Jun and c-Fos) was performed by Western blot analysis using specific antibodies. Relative protein levels were normalized to control protein levels; and (**C**) Detection of STAT-1 was performed by Western blot analysis using specific antibodies. Relative protein levels were normalized to control protein levels.

NF-κB is a major transcription factor that is activated during the inflammatory response to LPS. To determine whether pheophytin a affects the signaling pathways leading to NF-κB activation, cytosolic and nuclear extracts from LPS-stimulated RAW 264.7 cells were prepared. Pheophytin a had no effect on LPS-induced cytosol and nuclear localization and phosphorylation of NF-κB p65 (Figure 5A). Inflammatory mediators were mediated not only by NF-κB but also by other transcription factors, such as AP-1. Following inflammatory stimulation, the AP-1 heterodimer, c-Jun and c-Fos translocated into the nucleus, leading to the transcription of several inflammatory proteins. To determine whether AP-1 is involved in the inhibition of these inflammatory factors by pheophytin a, the nuclear levels of c-Jun and c-Fos were examined. After LPS stimulation, both c-Jun and c-Fos were significantly induced. Pheophytin a pretreatment did not reduce the quantities of c-Jun and c-Fos in a time-dependent manner (Figure 5B). STAT-1 has been implicated to play roles in inflammatory signaling cascades triggered by LPS [15,27]. To assess whether the inhibitory effect of pheophytin a on the expression of pro-inflammatory cytokines and mediators is mediated via STAT-1, we first examined the effect of pheophytin a on LPS-induced STAT-1 activation. As shown in Figure 5C, enhanced STAT-1 phosphorylation was prevented by pheophytin a treatment in a time-dependent manner in RAW264.7 cells stimulated with LPS. Taken together, our data indicate that pheophytin a suppresses LPS-induced inflammatory responses through inhibiting STAT-1 activation in RAW264.7 macrophages.

## 3. Experimental Section

### 3.1. The Purification of Pheophytin a

The leaves of *Nelumbo nucifera* Gaertn. cv*. Rosa-plena* were collected from Tainan County, Taiwan, November 2008. Plant material was identified by Dr. Fu-Yuan Lu (Department of Forestry and Natural Resources College of Agriculture, National Chiayi University, Chiayi, Taiwain). A voucher specimen (*Nelumbo nucifera* Gaertn. cv. *Rosa-plena*) was deposited in the School of Medical and Health Sciences, Fooyin University, Kaohsiung, Taiwan. The air-dried leaves of *Nelumbo nucifera* Gaertn. cv. *Rosa-plena* (1.5 kg) were extracted with MeOH (50 L × 5) at room temperature and a MeOH extract (108.7 g) was obtained upon concentration under reduced pressure. The MeOH extract, suspended in H_2_O (1 L), was partitioned with CHCl_3_ (3 L × 4) to give fractions soluble in CHCl_3_ (57.2 g) and H_2_O (43.6 g). The CHCl_3_-soluble fraction was chromatographed over silica gel (1700 g, 70–230 mesh) using *n*-hexane/EtOAc/MeOH mixtures as eluents to produce six fractions. Part of fraction 3 (7.83 g) was subjected to silica gel chromatography, by eluting with n-hexane-Acetone (7:1), enriched gradually with Acetone, to furnish two fractions (3-1–3-2). Fraction 3-1 (4.73 g) was further purified on a silica gel column using *n*-hexane/Acetone mixtures to obtain β-sitostenone (9 mg) and stigmasta-4,22-dien-3-one (8 mg) and pheophytin a (32 mg). The structure of pheophytin a was identified by spectroscopic analysis (purity > 95%).

### 3.2. The Chemical Structure and Purity of Pheophytin a

Pheophytin a, C_55_H_74_N_4_O_5_, Deep green needles (CHCl_3_), mp 113–114 °C, UV λ_max_ 229, 274, 330, 372, 406, 508, 540, 610, 665 nm, IR ν_max_ 1620, 1699, 1740, 3400 cm^−1^, ^1^H NMR (400 MHz, CDCl_3_): δ-1.62 (1H, br s, NH, D_2_O exchangeable), 0.81, 0.83 (each 3H, *d*, *J* = 6.6 Hz, H-38, 39), 0.87 (6H, *d*, *J* = 6.6 Hz, H-36, 37), 1.60 (3H, *s*, H-40), 1.01–1.10 (21H, *m*, H-24–35), 1.64 (3H, *t*, *J* = 7.6 Hz, H-8^2^), 2.22 (1H, *m*), 2.32 (1H, *m*), 2.54 (1H, *m*), 2.65 (1H, *m*), 3.22, 3.42 (each 3H, *s*, H-7^1^, 2^1^), 3.54 (2H, *q*, *J* = 7.6 Hz, H-8^1^), 3.72, 3.88 (each 3H, *s*, H-12^1^, OCH_3_), 4.22 (1H, *m*, H-17), 4.48 (2H, *m*, H-18), 4.49 (1H, *d*, *J* = 7.2 Hz, H-21), 5.16 (1H, *t*, *J* = 7.4 Hz, H-22), 6.19 (1H, *d*, *J* = 11.6 Hz, H-3^2^), 6.30 (1H, *d*, *J* = 17.8 Hz, H-3^2^), 6.28 (1H, *s*, H-13^2^), 8.00 (1H, *dd*, *J* = 17.8, 11.4 Hz, H-3^1^), 8.57, 9.39, 9.52 (each 1H, *s*, H-20, 5, 10), EI-MS *m*/*z*: 871 [M]^+^.

### 3.3. Reagents and Antibodies

LPS (L2654) was purchased from Sigma (St. Louis, MO, USA). The NOS2 antibody was purchased from BD Biosciences (San Jose, CA, USA). Antibodies against phospho-IκB-α (Ser32/36), p38 MAP Kinase (Thr180/Tyr182), ERK1/2 MAP Kinase (Thr202/Tyr204), SAPK/JNK (Thr183/Tyr185), Akt (Ser473), and STAT1 (Tyr701) were all purchased from Cell Signaling (Danvers, MA, USA). All other chemicals were of the purest analytical grade and were purchased from Sigma.

### 3.4. Cell Culture

RAW 264.7 cells, derived from murine macrophages, were obtained from the Bioresource Collection and Research Center (BCRC 60001, Hsinchu, Taiwain). RAW 264.7 cells were cultured in DMEM (with 10% heat-inactivated fetal bovine serum, 100 U/mL penicillin, 100 μg/mL streptomycin, 25 g/mL amphotericin B, 2 mM l-glutamine, were all purchased from Biological Industries (Kibbutz Beit Haemek, Israel).

### 3.5. Cytotoxicity of Pheophytin a

Pheophytin a. cytotoxicity was evaluated using the Alamar Blue assay. In brief, 200 ml 10% Alamar Blue reagent (Serotec Ltd. Scandinavia, Hamar, Norway) was added into a medium containing 2 × 10^4^ cells/200 μL/well in which the cells were treated with 5, 10, and 25 μM of pheophytin a. Twenty-four hours later, the colorimetric readings were evaluated with an ELISA plate reader at 570 and 600 nm to compare the cytotoxicity of pheophytin a. treatment with controls.

### 3.6. Pheophytin a Treatment and LPS Stimulation of RAW 264.7 Cells

RAW 264.7 cells were transferred to 2% fetal bovine serum for 3 h for serum starvation (as activation). We then treated the RAW 264.7 cells with the indicated concentrations of pheophytin a. Thirty minutes later, we stimulated the RAW 264.7 cells with LPS (100 ng/mL). Three hours after the LPS stimulation, we collected the cells to measure RNA expression levels. Six hours after LPS stimulation, we collected the cells to measure protein production.

### 3.7. Measurement of Nitrite Release

Nitrite (a stable metabolite of NO) concentration in the supernatant was measured as an indicator of NO production using the Griess Reagent System (Promega Biotech Co., Ltd., Madison, WI, USA) according to the manufacturer’s protocols. In brief, cells were treated with various doses of pheophytin a and LPS. At the end of the reaction, 50 μL of each supernatant was carefully transferred into a 96-well plate, with the subsequent addition of the Griess reagent. After color development for 10 min, absorbance was measured on the reader at a wavelength of 520 nm. This absorbance was normalized using a standard curve to obtain the nitrite concentration.

### 3.8. Western Blot Measurement of the Protein Levels of NOS2 and COX-2

RAW 264.7 cells were pre-treated with different concentrations of pheophytin a, and the supernatants were collected 6 h later, after cell stimulation with LPS. NOS2 and COX-2 protein expression levels were measured by Western blot.

### 3.9. Real-Time Quantitative PCR

For mRNA measurement, we first extracted mRNA from RAW 264.7 cells using the RNAspin Mini RNA Isolation Kit (GE Healthcare, Buckinghamshire, UK). We then performed real-time quantitative PCR in a Roche LightCycler (Mannheim, Germany) to measure NOS2 and COX-2 mRNA levels.

### 3.10. NOS2 Promoter Activity Assay

We used a Luciferase assay system (Promega) to detect NOS2 promoter activity according to the manufacturer’s protocol. Briefly, we removed the culture medium and washed with PBS 3 times. We then added reporter lysis 5× buffer (Promega) for 20 min at room temperature and froze the specimens at −80 °C for another 20 min. Finally, we examined the specimen (at room temperature) by luminometer and ELISA reader to perform the luciferase (Promega) and β-galactosidase assays.

### 3.11. NF-κB Translocation Assay

We then investigated whether the pheophytin a inhibitory effect on NO production resulted from the inhibition of NF-κB translocation into the nucleus. We harvested cytosol and nuclear proteins 15, 30, 60, and 120 min after LPS stimulation. Buffer A (10 mM HEPES (pH 7.9), 10 mM KCl, 1.5 mM MgCl_2_, 10 mM DTT, 4% 25× PI (protease inhibitors), and 0.375% NP-40 in ddH_2_O) and buffer C (20 mM HEPES, 400 mM NaCl, 1 mM EDTA, 1 mM EGTA, 10 mM DTT, and 4% 25× protease inhibitors in ddH_2_O) were prepared immediately before use. In brief, the supernatant was removed and followed by two washings of 1× PBS. Three hundred microliters of buffer A was then added into each dish, ensuring that the cells were completely covered. Ten minutes later, the edges of the dish were knocked to let cells shed from the bottom of dish, and a scraper was used to collect cells. After 10-s vortex mixing, the cytosol extract was prepared by 1-min centrifugation at 10,000× *g*; the resulting pellet contained the nuclear extract. The pellet was washed twice with 1× PBS, 100 μL of buffer C was added into each sample, and the nuclear envelope was broken by stirring with a stir bar for 20 min. Nuclear extracts were prepared by 10-min centrifugation at 10,000× *g*. All of the above procedures were performed on ice.

### 3.12. Enzyme-Linked Immunosorbent Assay (ELISA)

RAW 264.7 cells were plated at a density of 4 × 10^5^ cells per well in 12-well plates. Cells were pre-treated with different concentrations of pheophytin a, and the supernatants were collected 6 h later, after cell stimulation with LPS. The quantities of PGE2, COX-2, and IL-1β proteins were measured using ELISA kits for PGE2 (R&D Systems, Minneapolis, MN, USA), COX-2 (R&D Systems, Minneapolis, MN, USA), and IL-1β (eBioscience, San Diego, CA, USA), respectively. Each treatment was performed in duplicate wells, and triplicate experiments were performed.

### 3.13. Signal Transduction Pathway Assay

We performed Western blot analysis on the translocation of the components of the NF-κB pathway (IkBα, p65) and the phosphorylation of ERK1/2, p38, JNK, Akt, and STAT-1 proteins induced by LPS in RAW 264.7 cells. Cells were pre-treated with pheophytin a for 30 min, and both total and nuclear proteins were harvested at three different time points (10, 15, and 30 min) after stimulation with LPS (100 ng/mL). These experiments were repeated three times. The ERK1/2 chemical inhibitor U0126 was also used to confirm the role of ERK1/2 induced by pheophytin a. We added U0126 before pheophytin a treatment on RAW 264.7 cells.

### 3.14. Statistical Analysis

All results are expressed as the means ± standard deviations (SD), with *n* indicating the number of experiments. Statistical significance was determined by Student’s *t*-test for two points. All differences were considered significant at a *p* value of <0.05.

## 4. Conclusions

In this study, we demonstrate that pheophytin a can suppress NO production in LPS-stimulated RAW 264.7 cells, possibly due to dual mechanisms of inhibiting NOS2 promoter activities (and hence reducing *NOS2* gene expression and NOS2 protein production) and modulating the ERK and STAT-1 pathway. Pheophytin a also had a protective effect because of the attenuation of PGE2 and pro-inflammatory cytokine IL-1β production. We provide evidence to suggest that pheophytin a has effective anti-inflammatory properties that have the potential to improve the outcome of endotoxin sepsis in the future.

NO is a signaling molecule that has multiple physiological effects on various organ systems, such as host defense, inflammation and immune suppression [28]. We found that pheophytin a showed very strong inhibitory activity against NO production in LPS-stimulated RAW 264.7 cells. Three isoforms of NOS, including endothelial nitric oxide synthase (eNOS), neuronal nitric oxide synthase (nNOS) and iNOS have been identified in mammalian cells based on their physical and biochemical characteristics. iNOS is overexpressed in various cell types, including macrophages, hepatocytes, and astrocytes, in response to immunomodulating molecules, such as LPS, IL-1β and pro-inflammatory cytokines [28,29]. During sepsis, NOS2, a key enzyme catalyzing the dramatic increase in NO by LPS, plays an important role in the pathophysiology of endotoxemia and sepsis [30].

PGE2, which is converted from LPS-induced endogenous arachidonic acid via COX-2-catalyzed reactions, is considered a principal mediator of inflammation [21,31]. Non-steroidal anti-inflammatory drugs (NSAIDs) are considered to diminish the inflammation seen in many inflammatory diseases by reducing PGE2 production [32]. Considering that pheophytin a possesses the potential for inhibiting LPS-induced NO production, Pheophytin a might be used as an anti-inflammatory lead compound for NSAID development or as a functional food additive for the prevention and treatment of inflammation.

MAPKs involved in macrophage inflammation, including ERK1/2, JNK and p38 MAPK, play important regulatory roles in controlling cellular responses to inflammatory cytokines [25]. The MAPK family plays an important role in the LPS-induced expression of iNOS, COX-2, and proinflammatory cytokines in many cell types [26,33]. Several natural products inhibit the expression of these genes by modulating MAPK phosphorylation. In the current study, LPS induced the phosphorylation of ERK1/2 in RAW264.7 cells in the absence of pheophytin a. Pheophytin a modulated LPS-induced ERK1/2 production, with the response being reversed by the ERK1/2 inhibitor U0126. These results suggest that pheophytin a exerts anti-inflammatory effects by regulating specific MAPKs. Although we do not have direct evidence for the involvement of STAT1 and ERK1/2 in pheophytin a-induced protection at this time, we can logically speculate on the participation of these immunoprotective signaling mediators. Further studies are needed to prove this assumption.

STATs play roles in LPS-triggered inflammatory signaling cascades [34]. Several studies have demonstrated that STAT signaling is essential for LPS-induced iNOS expression in RAW264.7 macrophages [35,36]. Pheophytin a dramatically suppressed LPS-induced phosphorylation of STAT-1, suggesting that it preferentially inhibits STAT signaling, leading to down-regulation of iNOS expression in LPS-inflamed macrophages.

**Figure 6:**
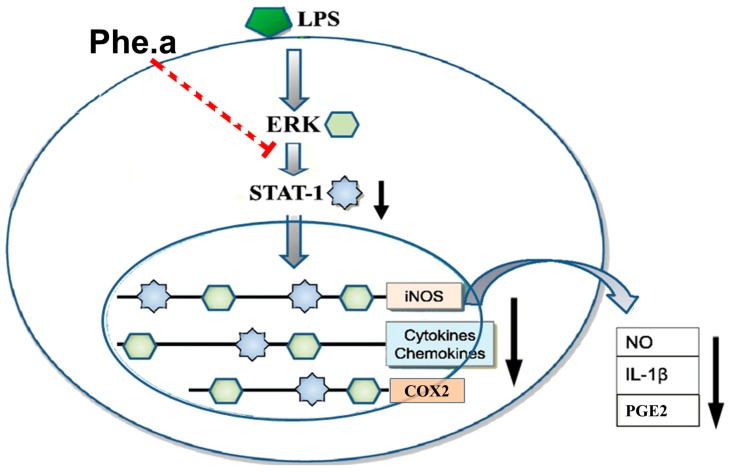
A schematic representation of the suppression of LPS-induced NO, PGE2, and IL-1β production using pheophytin a (Phe. a), through the inhibition of the ERK1/2-STAT-1 pathway. Red dotted line represents that pheophytin a inhibits ERK1/2-STAT-1 pathway. Black arrow represents the reduction of NO, PGE2 and IL-1β production.

In conclusion, pheophytin a showed potent anti-inflammatory activity by inhibiting NO production in LPS-stimulated RAW 264.7 cells. We also identified a potential mechanism to explain the anti-inflammatory activity of pheophytin a (Figure 6). Hence, these results suggest that pheophytin a possesses potential anti-inflammatory activity and holds great promise for the treatment of inflammatory diseases.
